# Early life microbiome perturbation alters pulmonary responses to ozone in male mice

**DOI:** 10.14814/phy2.14290

**Published:** 2020-01-24

**Authors:** Traci A. Brown, Hiroki Tashiro, David I. Kasahara, Youngji Cho, Stephanie A. Shore

**Affiliations:** ^1^ Molecular and Integrative Physiological Sciences Program Department of Environmental Health Harvard T.H. Chan School of Public Health Boston MA USA

**Keywords:** 16S rRNA sequencing, airway responsiveness, neutrophil, sex differences

## Abstract

Early life changes in the microbiome contribute to the development of allergic asthma, but little is known about the importance of the microbiome for other forms of asthma. Ozone is a nonatopic asthma trigger that causes airway hyperresponsiveness and neutrophil recruitment to the lungs. The purpose of this study was to test the hypothesis that early life perturbations in the gut microbiome influence subsequent responses to ozone. To that end, we placed weanling mouse pups from The Jackson Laboratories or from Taconic Farms in sex‐specific cages either with other mice from the same vendor (same‐housed) or with mice from the opposite vendor (cohoused). Mice were maintained with these cagemates until use. The gut microbial community differs in mice from Jackson Labs and Taconic Farms, and cohousing mice transfers fecal microbiota from one mouse to another. Indeed, 16S rRNA sequencing of fecal DNA indicated that differences in the gut microbiomes of Jackson and Taconic same‐housed mice were largely abolished when the mice were cohoused. At 10–12 weeks of age, mice were exposed to room air or ozone (2 ppm for 3 hr). Compared to same‐housed mice, cohoused male but not female mice had reduced ozone‐induced airway hyperresponsiveness and reduced ozone‐induced increases in bronchoalveolar lavage neutrophils. Ozone‐induced airway hyperresponsiveness was greater in male than in female mice and the sex difference was largely abolished in cohoused mice. The data indicate a role for early life microbial perturbations in pulmonary responses to a nonallergic asthma trigger.

## Graphical abstract

We altered the gut microbiomes of weanling mice by placing them in cages occupied by mice from another vendor. Our data indicate that in male mice, early life microbial perturbations alter the pulmonary response to ozone.

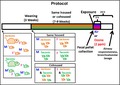

## 1 INTRODUCTION

Ozone is a trigger for asthma. After days during which ambient ozone concentrations are high, emergency room visits and hospitalizations for asthma increase (Fauroux, Sampil, Quénel, & Lemoullec, 2000). In human studies, ozone exposure causes asthma symptoms (Goodman et al., 2018), decreases in pulmonary function (Yang et al., 2005), and also causes airway hyperresponsiveness (AHR), a characteristic feature of asthma (Foster, Brown, Macri, & Mitchell, 2000). Ozone recruits neutrophils to the airways and increases the production of acute phase cytokines and chemokines (Balmes et al., 1997). Similar to human subjects, mice also exhibit AHR and increases in bronchoalveolar lavage (BAL) neutrophils after acute ozone exposure (Cho, Zhang, & Kleeberger, 2001; Zhang, Levitt, & Kleeberger, 1995).

The microbiome is a complex community of microorganisms found in many parts of the body, with the greatest number in the gut. An increasing body of evidence indicates that the microbiome influences health and disease states. For example, the microbiome has been shown to play a role in Crohn's disease, as well as in several autoimmune and neurological disorders (Austin, Mellow, & Tierney, 2014; Cojocaru & Chicoş, 2014; Ghaisas, Maher, & Kanthasamy, 2016; Øyri, Műzes, & Sipos, 2015). Early life is a critical period for gut colonization with bacteria, and perturbations in the gut microbiome during this time can impact physiological systems which are maturing simultaneously (Zeissig & Blumberg, 2014). For example, in mice, early exposure to antibiotics shifts the Th1/Th2 immune system balance toward a more Th2 dominant profile, including increase in production of interleukin‐4 and IgE (Oyama, Sudo, Sogawa, & Kubo, 2001). Early life antibiotic treatment also alters metabolism of carbohydrates, lipids, and cholesterol later in life (Cho et al., 2012). In humans, early exposure to antibiotics increases the risk of obesity, consistent with a role for the microbiome in metabolism (Arrieta et al., 2015).

There is increasing evidence that the microbiome also plays a role in the development of asthma. For example, there are differences in both the gut and lung microbiomes in subjects with asthma (Arrieta et al., 2015; Durack et al., 2018; Hilty et al., 2010; Huang et al., 2011; Kilkkinen et al., 2006; van Nimwegen et al., 2011; Stokholm et al., 2018). Importantly, early life events that alter the developing gut microbiome (early exposure to antibiotics, formula vs. breastfeeding, cesarean vs. vaginal delivery, exposure to pets or farm animals) also affect the risk of allergic asthma (Arrieta & Finlay, 2014). Furthermore, several studies have reported that early life changes in the microbiome are predictive of subsequent asthma development (Arrieta et al., 2015; Durack et al., 2018; Stokholm et al., 2018).

Data from mice also support a role for the microbiome in asthma. In mouse models of allergic asthma, treatment with certain antibiotics or germ‐free (GF) conditions exacerbate allergic airway responses (Arnold et al., 2011; Herbst et al., 2011; Olszak et al., 2012; Russell et al., 2012). Moreover, as in the human studies, the early life window is important for these microbiome‐dependent changes. Reconstituting GF mice by fecal transfer from conventional mice attenuates allergic airway responses only when the transfer is performed early in life (Olszak et al., 2012). Similarly, in mouse models of allergen sensitization and challenge, perinatal but not adult antibiotic treatment of mice exacerbates allergic airways disease (Russell et al., 2013, 2012). In addition, early life but not adult inoculation with certain bacterial species (Nunes et al., 2018), including some identified as protective against asthma in children (Arrieta et al., 2015), reduces serum IgE, a marker of allergic sensitization.

Previous studies indicate that the microbiome may also play a role in nonallergic forms of asthma. In male mice, the magnitude of AHR induced by ozone exposure is decreased by both antibiotic treatment and GF conditions (Cho et al., 2018). The magnitude of ozone‐induced AHR is greater in male than in female mice (Birukova et al., 2019; Cho, Abu‐Ali, et al., 2019; Kasahara et al., 2019), and this sex difference is abolished when mice are treated with antibiotics (Cho, Abu‐Ali, et al., 2019). Furthermore, when weanling female pups are housed in cages conditioned by adult male mice, they develop greater ozone‐induced AHR than weanling female pups raised in cages conditioned by adult females (Cho, Abu‐Ali, et al., 2019).

The purpose of this study was to examine the hypothesis that early life perturbations of the microbiome in mice alter responses to ozone later in life. To do so, we purchased pregnant C57BL/6 mice from The Jackson Laboratories and from Taconic Farms. At weaning, pups were placed in sex‐specific cages either with other mice from the same vendor (same‐housed) or with mice from the opposite vendor (cohoused). Mice were maintained with these cagemates until use. The gut microbial community structures differ in mice from Jackson Labs and Taconic Farms (Ivanov et al., 2009; Velazquez et al., 2019). As a result of grooming and copraphagic behavior, cohousing mice transfers fecal microbiota from one mouse to another. When the mice reached early adulthood (10–12 weeks of age), they were exposed to room air or ozone (2 ppm for 3 hr). Pulmonary responses were evaluated 24 hr later. Our data indicated that compared to same‐housed mice, cohoused male but not female mice had reduced ozone‐induced AHR and reduced ozone‐induced increases in BAL neutrophils. The data indicate a role for early life microbial perturbations in pulmonary responses to a nonallergic asthma trigger.

## 2 METHODS

### 2.1 Mice

These studies were approved by the Harvard Medical Area Standing Committee on Animals. Pregnant female mice on a C57BL/6 background were purchased from two vendors, The Jackson Labs (Bar Harbor, ME) and Taconic Farms (Rensselaer, NY), and delivered, on day 14 of pregnancy, to the mouse vivarium at the specific pathogen free facility at the Harvard T.H. Chan School of Public Health. At weaning, the offspring were placed on LabDiet 5053 from PicoLab. In the vivarium, lights went on at 6 am and off at 6 pm Note that although the C57BL/6 mice from Jackson Labs and Taconic Farms are genetically similar, they are not genetically identical due to genetic drift that has occurred over the years since the colonies diverged (Mekada et al., 2009).

### 2.2 Protocol

**Figure 1:**
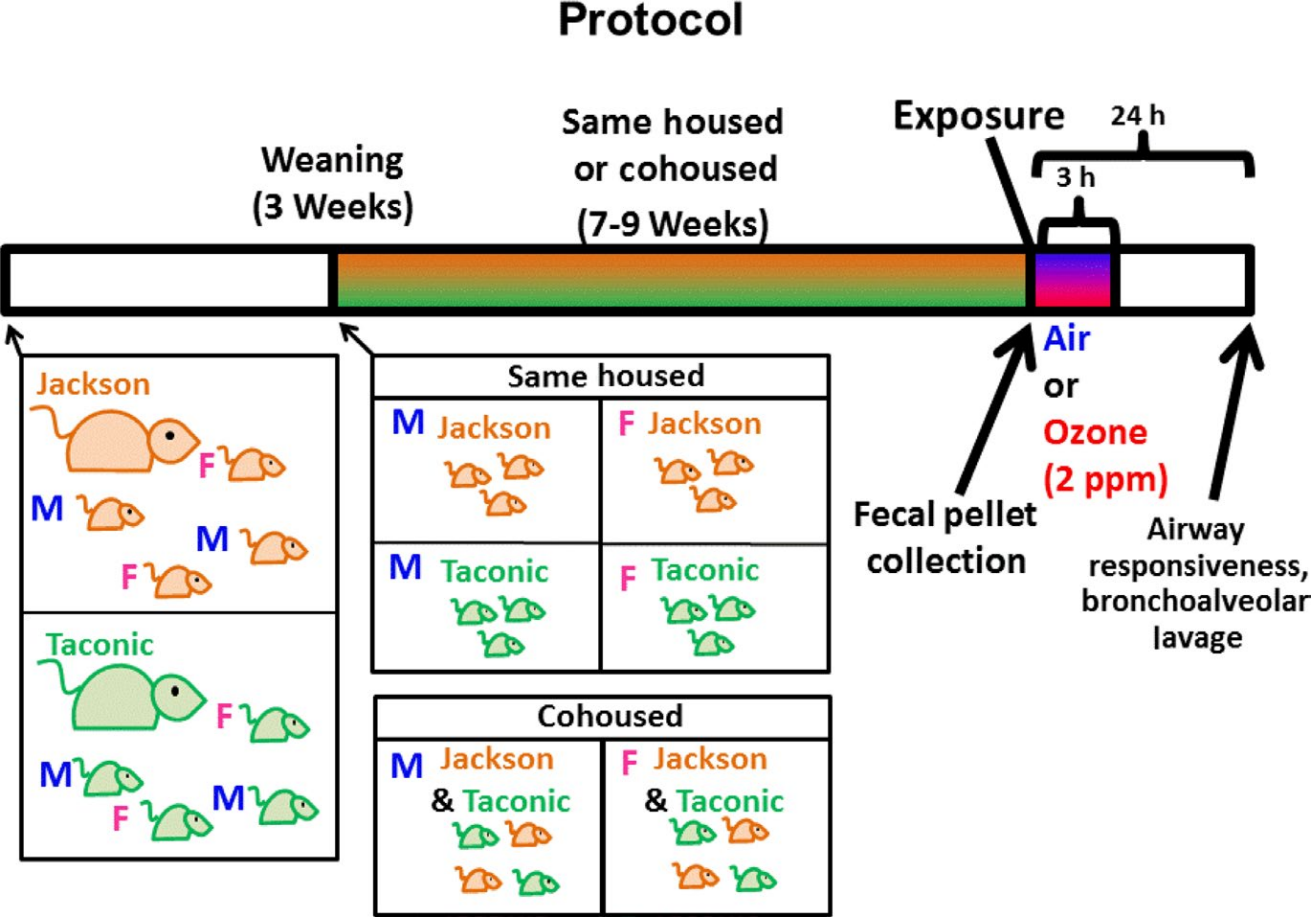
Study Protocol. Pregnant C57BL/6 mice were obtained from The Jackson Labs and from Taconic Farms. Pups were born at HSPH and then weaned at 3 weeks of age. At weaning, pups were put in a cage with other pups from a different mother but from the same vendor as their own mother (same‐housed) or with pups from a mother from the other vendor (cohoused). Pups were housed for an additional 7–9 weeks before being exposed to air or ozone. A fecal pellet was collected prior to air or ozone exposure to evaluate the taxonomic composition of the gut microbiome. At 24 hr after air or ozone exposure, airway responsiveness was determined and a bronchoalveolar lavage was performed

The experimental protocol is outlined in Figure 1. At weaning (approximately 3 weeks of age), pups were separated by sex and put into cages with other pups from a different mother but from the same vendor (same‐housed) or into cages with pups from a different mother from the other vendor (cohoused). Most mice (about 80%, 35 out of 43 total cages) were housed with four pups per cage, with the majority of those being two pups from one mother paired with two pups from another mother (either from the same vendor or the other vendor). The remaining mice were housed in a 3:2 ratio (4 cages), a 2:1 ratio (2 cages), a 1:1 ratio (1 cage), or housed alone (1 cage). Pups remained with these cagemates for an additional 7–9 weeks before being exposed to air or ozone (2 ppm) for 3 hr. A fecal pellet was collected from each mouse prior to exposure to determine the taxonomic composition of the gut microbiome. Twenty‐four hours after air or ozone exposure, lung mechanics and airway responsiveness to inhaled aerosolized methacholine chloride were determined. Mice were euthanized immediately after these measurements were completed and BAL was performed. Data presented are from three cohorts of mice.

### 2.3 Ozone exposure

Mice were exposed for 3 hr to room air or to ozone (2 ppm) in stainless steel and plexiglass exposure chambers as previously described (Shore, Rivera‐Sanchez, Schwartzman, & Johnston, 2003). During exposure, mice were placed within individual wire mesh cages and food and water were withdrawn. Immediately after exposure, mice were placed in fresh cages, individually housed, and food and water were restored. Ozone exposure was typically begun between 9 and 10 am.

### 2.4 Pulmonary mechanics and airway responsiveness

Mice were anesthetized, intubated with a tubing adaptor, and ventilated (Flexivent, Scireq) as previously described (Williams et al., 2013) except that the chest wall was not opened. A positive end expiratory pressure of 3 cm H_2_O was then applied by placing the expiratory line under water. Three volume excursions to total lung capacity (TLC) (30 cm H_2_O trans‐respiratory system pressure), each separated by 1 min, were first administered to establish a common volume history. The following sequence was then initiated. One minute after an excursion to TLC, 10 breaths from an aerosol generated from PBS were administered. Then, total respiratory system resistance (Rrs) was measured every 15 s for the next 3 min using the forced oscillation technique. This sequence was then repeated with aerosolized methacholine chloride increasing in half‐log concentrations from 1 to 100 mg/ml. The three highest values of R_RS_ obtained after each dose were averaged to construct dose–response curves.

### 2.5 Bronchoalveolar lavage

The lungs were lavaged twice with 1 ml of ice‐cold PBS. These two lavages were pooled and centrifuged at 400*g* at 4°C for 10 min. The supernatant was stored at −80°C until further analysis. The cell pellet was resuspended in PBS and total cell numbers were counted using a hemocytometer. Aliquots of cells were also centrifuged onto glass slides (Cytospin 3, Shandon), air‐dried overnight, and stained with Wright‐Giemsa (Hemacolor, Shandon). Cell differentials were determined by counting at least 300 cells under 400× magnification. Total BAL protein concentration was assessed using the BCA Protein assay (Pierce‐Thermo Fischer). A multiplex assay (Eve Technologies, Calgary) was used to assess cytokines and chemokines. Prior to analysis, BAL samples were concentrated approximately eightfold using an Amicon Ultra‐3 kDa filter (EMD‐Millipore) and concentrated by centrifugation. Values shown in figures are those in the original sample prior to concentration.

### 2.6 16S rRNA sequencing and analysis

Fecal pellets were harvested from each mouse on the day prior to ozone or air exposure and stored at −20°C. For extraction of fecal DNA, fecal pellets were incubated for 24 hr at 56°C with proteinase K. DNA was then isolated using QIAamp DNA Mini Kits (Qiagen) using ~25 mg of feces. Fecal DNA was submitted to the Massachusetts Host‐Microbiome Center at Brigham and Women's Hospital, and a multiplexed amplicon library covering the 16S rDNA gene V4 region was produced from the DNA samples on MiSeq (Illumina). Microbial analysis was performed using Qiime software with Greengene 99 database on the Nephele pipeline (Caporaso et al., 2010). This pipeline generates alpha and beta diversity and performs principle coordinate analysis (PCoA) calculated using Bray–Curtis distances. Sequence reads were assigned to each phylum and genus at 99% sequence similarity cutoff. The Multivariate Association with Linear Models (MaAsLin) statistical framework on Galaxy module was used to assess differential taxonomy abundance and association with experimental variables (Morgan et al., 2012). For each microbial taxon, MaAsLin fits a linear model using arcsine square root transformed feature abundance (variance stabilizing transformation). Multiple hypothesis tests were adjusted to produce a final Benjamini and Hochberg false discovery rate and associations that had *q*‐values <.25 were considered to be significant. The unprocessed sequencing data is available at http://www.ncbi.nlm.nih.gov/bioproject/559353. The BioProject ID is PRJNA559353.

### 2.7 Microbial analysis via PCR

To compare the relative abundances of various bacterial taxa in fecal DNA from female mice, we used qPCR analysis quantified by SYBR green and normalized the data to total bacterial abundance using pan‐bacterial primers. Primer sequences used for this PCR were: pan‐bacterial forward: 5’‐AAACTCAAKGAATTGACGG K = G or T, reverse: 5’‐CTCACRRCGAGCTGA, R = A or G (Bacchetti De Gregoris, Aldred, Clare, & Burgess, 2011); Firmicutes phylum: forward: 5’‐GGAGYATGTGGTTTAATTCGAAGCA (Y = T/C), reverse: 5’‐AGCTGACGACAACCATGCAC; Bacteriodetes phylum: forward: 5’‐CRAACAGGATTAGATACCCT (R = G/A) reverse: 5’‐GGTAAGGTTCCTCGCGTAT; γ‐Proteobacteria forward: 5’‐TCGTCAGCTCGTGTYGTGA (Y = T/C), reverse: 5’‐CGTAAGGGCCATGATG; and *Turicibacter* genus: forward: 5’‐CAGACGGGGACAACATTGGA, reverse: 5’‐TACGCATCGTCGCCTTGGTA.

### 2.8 Statistics

Except for analysis of 16S sequencing data (see above), the significance of differences between groups was assessed using factorial ANOVA (Statistica Software) using vendor, housing, and exposure as main effects. Fisher's LSD test was used for post hoc analysis. Male and female mice were analyzed separately. For BAL cells, in order to conform to a normal distribution, data were log‐transformed prior to analysis. A *p*‐value <.05 (two‐tailed) was considered significant. All values are expressed as mean ± *SEM*.

## 3 RESULTS

### 3.1 Body weights

Factorial ANOVA indicated no effect of either vendor or housing on body mass in male mice. In female mice, there was an effect of cohousing. Follow‐up analysis indicated that the effect lay in the Taconic female mice, in which the cohoused mice had significantly lower body mass than the same‐housed mice (Table 1).

**Table 1:** Body weights measured prior to exposure

	Male	Female
Group	Weight (g)	Weight (g)
Jackson same‐housed	27.8 ± 1.0 (16)	20.6 ± 0.3 (20)
Taconic same‐housed	28.3 ± 0.9 (15)	20.7 ± 0.3 (16)
Jackson cohoused	27.2 ± 0.9 (17)	20.3 ± 0.6 (15)
Taconic cohoused	26.0 ± 1.1 (15)	19.3 ± 0.6* (11)

Note

Results are mean ± *SE*. The number of mice in each group is indicated by the numbers in brackets. *p < .05 versus same‐housed mice from same vendor.

### 3.2 Pulmonary mechanics and airway responsiveness

#### 3.2.1 Innate airway responsiveness

**Figure 2:**
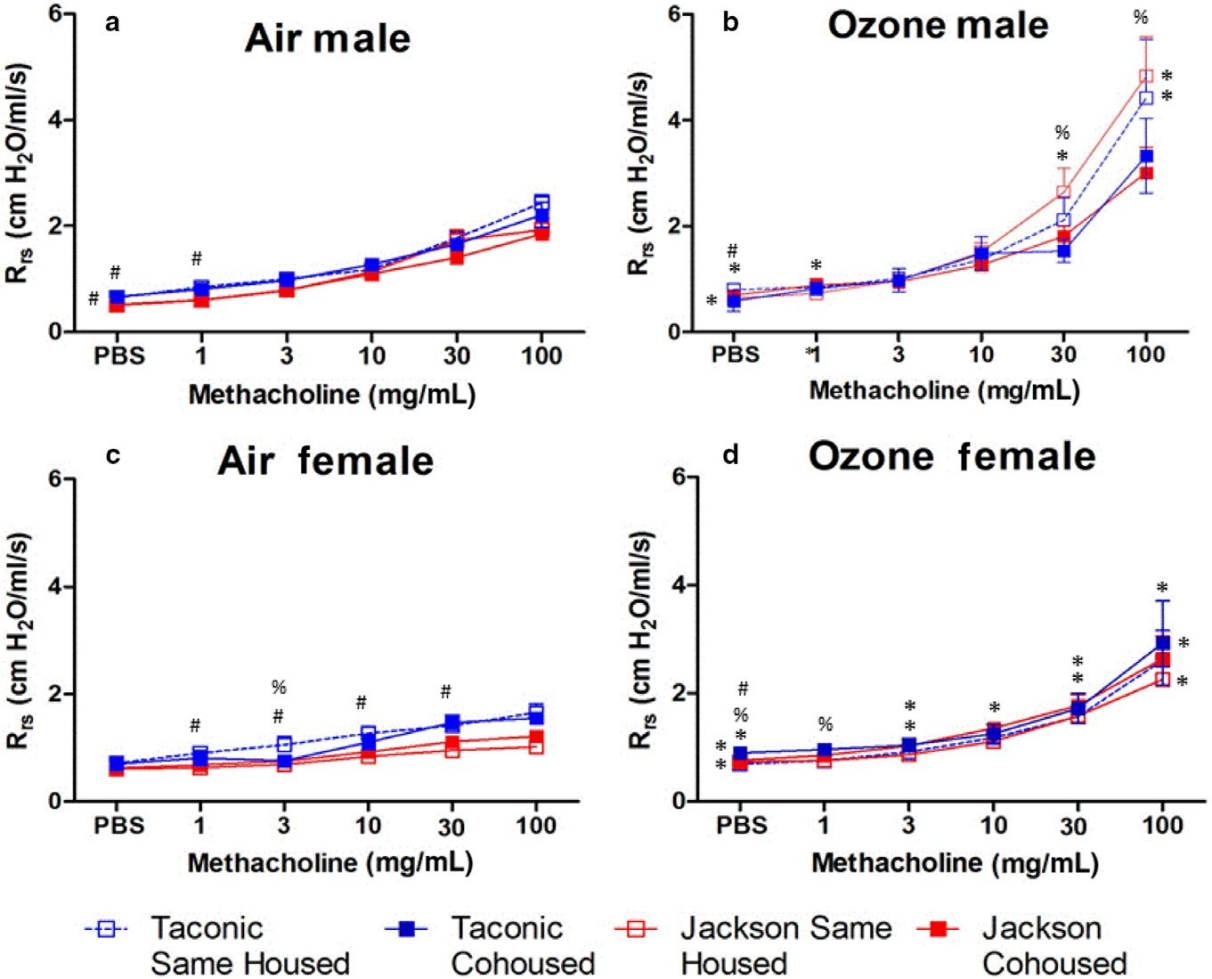
Airway responsiveness in mice exposed to air or ozone. Changes in pulmonary resistance (R_rs_) induced by inhaled aerosolized methacholine were assessed 24 hr after exposure to air (a,c) or ozone (b,d) in male (a,b) and female (c,d) mice. Results are mean ± *SE* of data from 5–10 mice/group, as indicated in Table 2. * *p* < .05 versus air‐exposed mice from the same vendor and housing group; # versus Jackson mice with same exposure and housing; % *p* < .05 versus cohoused mice from the same vendor and exposure group

In air‐exposed mice, factorial ANOVA indicated significantly greater baseline Rrs in Taconic than Jackson male mice (*p* < .01). A similar trend as observed in females but did not reach statistical significance. In males, there was no difference in airway responsiveness between Jackson and Taconic male mice whether the mice were same‐housed or cohoused (Figure 2a). However, airway responsiveness was greater in air‐exposed Taconic than Jackson female mice, particularly in the same‐housed mice (Figure 2c).

#### 3.2.2 Effects of ozone

In males, factorial ANOVA indicated that compared to air exposure, ozone exposure caused a small but significant (*p* < .001) increase in baseline Rrs. Follow‐up analysis indicated that this effect of ozone was only significant in the Jackson cohoused and the Taconic same‐housed mice (Table 2). In females, factorial ANOVA also indicated that compared to air exposure, ozone exposure caused a small but significant (*p* < .002) increase in baseline Rrs. Follow‐up analysis indicated that this effect of ozone was only significant in the Jackson same‐housed and the Taconic cohoused mice (Table 2).

**Table 2:** Baseline respiratory system resistance

	Group	Air	Ozone
		Rrs (cm H_2_O/ml/s)	Rrs (cm H_2_O/ml/s)
Male	Jackson same‐housed	0.51 ± 0.00 (7)	0.64 ± 0.03 (8)
	Jackson cohoused	0.52 ± 0.02 (6)	0.70 ± 0.04^*^ (6)
	Taconic same‐housed	0.64 ± 0.05^#^ (8)	0.80 ± 0.05^*#^ (9)
	Taconic cohoused	0.67 ± 0.04^#^ (9)	0.76 ± 0.09 (6)
Female	Jackson same‐housed	0.60 ± 0.02 (10)	0.73 ± 0.04^*^ (9)
	Jackson cohoused	0.62 ± 0.01 (6)	0.76 ± 0.04 (10)
	Taconic same‐housed	0.71 ± 0.05 (8)	0.69 ± 0.04^%^ (7)
	Taconic cohoused	0.7 ± 0.09 (8)	0.90 ± 0.08^*#^ (5)

Note

Results are mean ± *SE*. The number of mice in each group is indicated by the numbers in brackets. Rrs: respiratory system resistance; *p < .05 versus air exposed mice of same sex, housing, and vendor; ^#^ *p* < .05 versus Jackson mice of same sex, housing, and exposure; %*p* < .05 versus cohoused mice of same sex, exposure, and vendor.

In males, factorial ANOVA indicated that compared to air, ozone increased airway responsiveness (Figure 2b): at the two highest concentrations of methacholine, Rrs was significantly greater in ozone‐ than in air‐exposed mice. There was no difference in ozone‐induced AHR between Taconic mice and Jackson mice whether the mice were same‐housed or cohoused. However, there was a significant effect of cohousing on airway responsiveness. At the two highest concentrations of methacholine, Rrs was significantly greater in same‐housed than in cohoused mice. The effect reached statistical significance in the Jackson mice, though there was a similar trend in the Taconic mice. Indeed, in the cohoused mice, there was no significant effect of ozone versus air exposure except for baseline Rrs (PBS doses in Figure 2a and b, see Table 2).

In females, ozone also caused a significant increase in airway responsiveness (Figure 2d). As in the males, there was no difference in airway responsiveness in ozone‐exposed Jackson versus Taconic mice, whether the mice were same‐housed or cohoused. However, in contrast to the males, there was no effect of cohousing on airway responsiveness in ozone‐exposed females.

### 3.3 Pulmonary injury and inflammation

**Figure 3:**
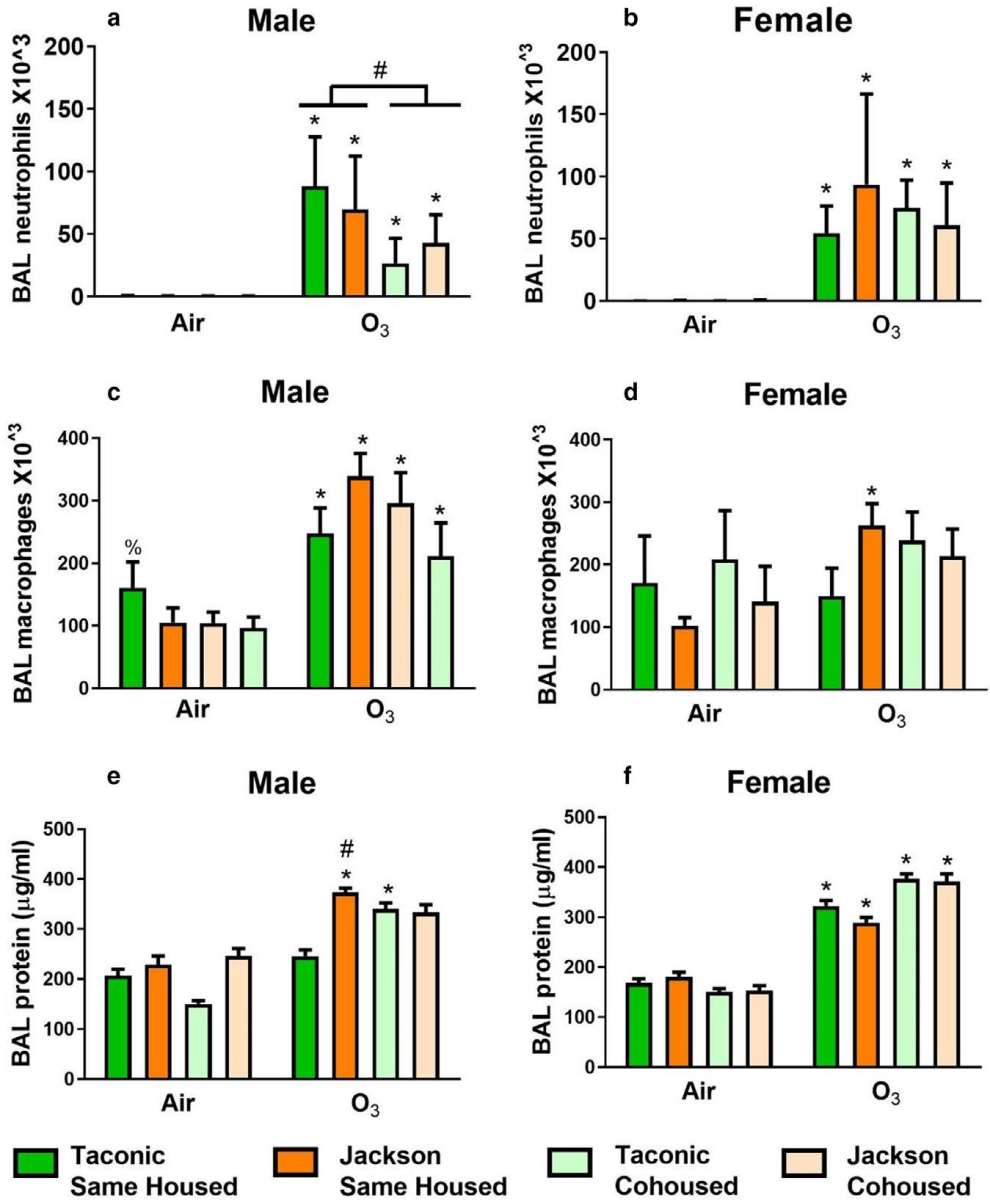
Pulmonary inflammation and injury in mice exposed to air or ozone. Bronchoalveolar lavage (BAL) neutrophils (a,b), macrophages (c,d), and protein (e,f) in male (a,c,e), and female (b,d,f) mice measured 24 hr after exposure to air or ozone (2 ppm for 3 hr). Results are mean ± *SE* of data from 4 to 10 mice/group for BAL cells and 6 to 12 mice/group for BAL protein. * *p* < .05 versus air‐exposed mice from the same vendor and housing group; % *p* < .05 for same‐housed mice versus cohoused mice; # versus Taconic mice with same exposure

Compared to air, ozone increased BAL neutrophils in all groups of mice, whether the mice were male or female (Figure 3). In males, there was no effect of vendor on BAL neutrophils whether the mice were same‐housed or cohoused. However, there was an effect of cohousing: compared to same‐housed male mice exposed to ozone, cohoused male mice had fewer BAL neutrophils (Figure 3a). In contrast, in female mice, there was no significant effect of vendor or cohousing on BAL neutrophils.

Compared to air, ozone also caused a significant increase in BAL macrophages in male mice, but there was no effect of either vendor or housing. There was also no significant effect of vendor or housing on BAL macrophages in ozone‐exposed female mice.

In males, exposure to ozone increased BAL protein in Jackson same‐housed and Taconic cohoused mice but not in the other groups of mice. BAL protein was significantly greater in same‐housed Jackson than Taconic mice exposed to ozone, but there was no significant difference in BAL protein in cohoused versus same housed mice from either vendor after ozone exposure. In females, exposure to ozone increased BAL protein in all four groups of mice, but there was no effect of vendor or housing on BAL protein.

**Figure 4:**
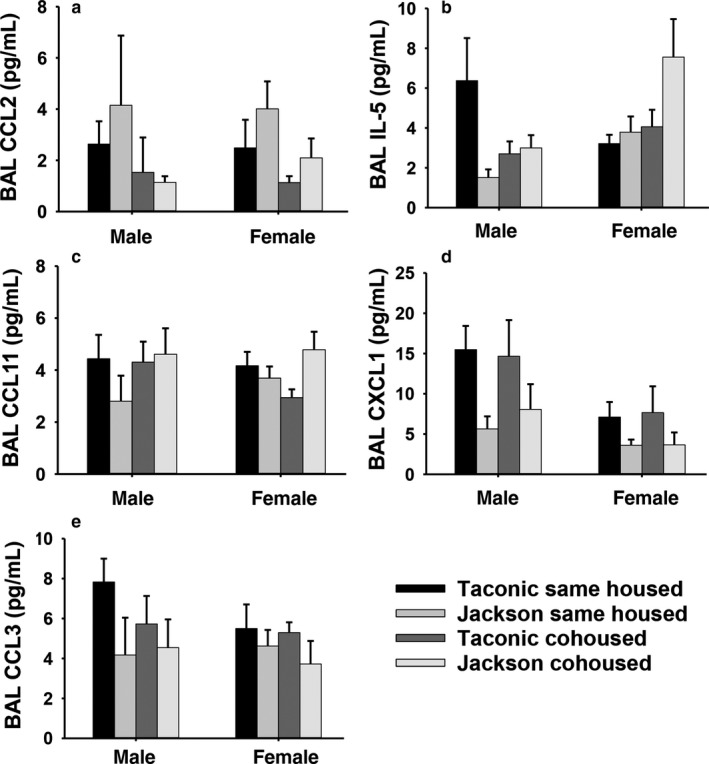
BAL concentrations of cytokines and chemokines in ozone‐exposed mice. BAL concentrations of CCL2 (A), IL‐5 (B), CCL11 (C), CXCL1 (D), and CCL3 (E) in male and female mice measured 24 hr after exposure ozone (2 ppm for 3 hr). Results are mean ± *SE* of data from 6 to 8 mice/group. Factorial ANOVA indicated significant (*p* < .05) effects of housing status for CCL2 and a significant interaction (*p* < .05) between housing status and vendor for IL‐5 and CCL11

Exposure to ozone increases BAL concentrations of many cytokines and chemokines that contribute to the neutrophil recruitment and AHR caused by O_3_ (Birukova et al., 2019; Cho, Abu‐Ali, et al., 2019; Cho et al., 2018; Kasahara et al., 2019; Mathews et al., 2017, 2018; Tashiro et al., 2019). To determine whether there were vendor‐ and/or housing‐related differences in the production of these inflammatory moieties following O_3_ exposure, we performed a multiplex assay (Figure 4). Among the various cytokines and chemokines measured, factorial ANOVA indicated a significant effect of housing status only for BAL concentrations of CCL2 (Figure 4a): BAL CCL2 was significantly lower in cohoused than same housed mice, similar to the effects of cohousing on airway responsiveness and neutrophil recruitment (Figures 2 and 3). However, there were also significant interactions between housing and vendor for CCL11 and IL‐5, although the direction of the change was opposite to the effect of housing on airway responsiveness and neutrophils: in Jackson but not Taconic mice, BAL CCL11, and BAL IL‐5 were significantly greater in cohoused than same housed mice (Figure 4b and c). Factorial ANOVA also indicated significant effects of vendor but not housing status on BAL CXCL1, and BAL CCL3 (Figure 4d and e). BAL CXCL1, and BAL CCL3 were both lower in Jackson than Taconic mice. Finally, there was an effect of sex for BAL CXCL1, and CXCL1 was significantly higher in males than in females (Figure 4b). There were no significant effects of housing, sex, or vendor on BAL G‐CSF, IL‐1α, IL‐6, LIF, CXCL2, or CXCL10.

### 3.4 16S rRNA sequencing

In male mice, cohousing of Taconic and Jackson mice from weaning attenuated pulmonary responses to ozone (Figures 2 and 3). Previous data from our laboratory indicate that it is the gut and not the lung microbiome that impacts pulmonary responses to ozone (Cho et al., 2018). Consequently, we performed 16S rRNA sequencing on fecal DNA from these male mice to determine how cohousing affected the gut microbiome.

**Figure 5:**
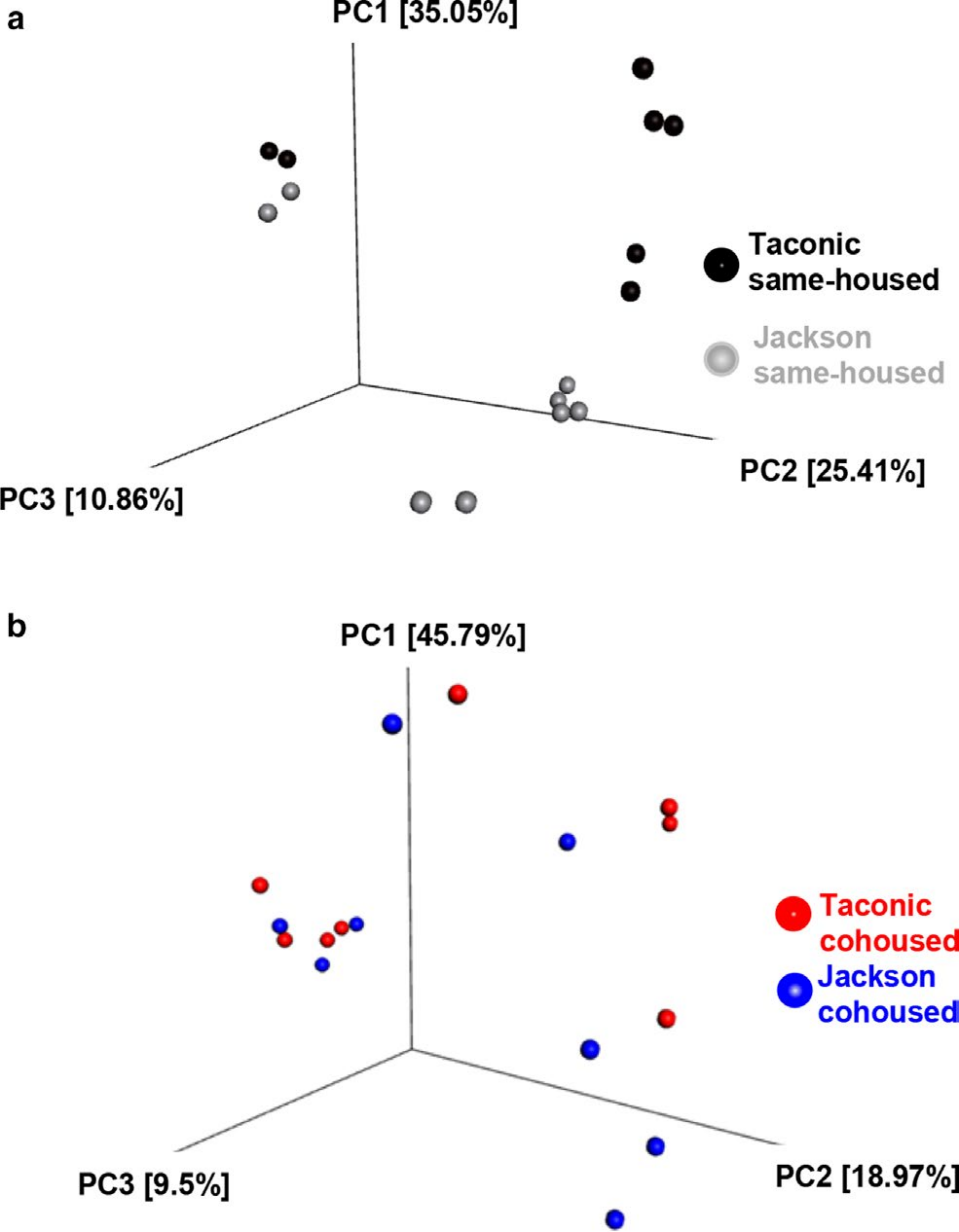
Differences in the gut microbiomes of Jackson and Taconic mice are attenuated by cohousing. Fecal pellets from 8 mice per group were harvested prior to exposure and 16S rRNA sequencing was performed on fecal DNA. (a) Principle coordinate analysis (PCoA) calculated by Bray–Curtis distances of same‐housed Jackson and Taconic mice; (b) PCoA of cohoused Jackson and Taconic mice

Principle coordinate analysis (PCoA) showed a clear separation between the gut microbial communities of Taconic and Jackson same‐housed male mice (Figure 5a), consistent with previous reports (Ivanov et al., 2009; Velazquez et al., 2019). However, in cohoused Taconic and Jackson mice, no such separation was observed (Figure 5b). The data indicate that differences in the gut microbiomes of Taconic and Jackson mice were substantially attenuated after cohousing mice from these two vendors together. There were no significant differences in diversity or richness between Taconic and Jackson mice whether the mice were same‐housed or cohoused (data not shown).

**Figure 6:**
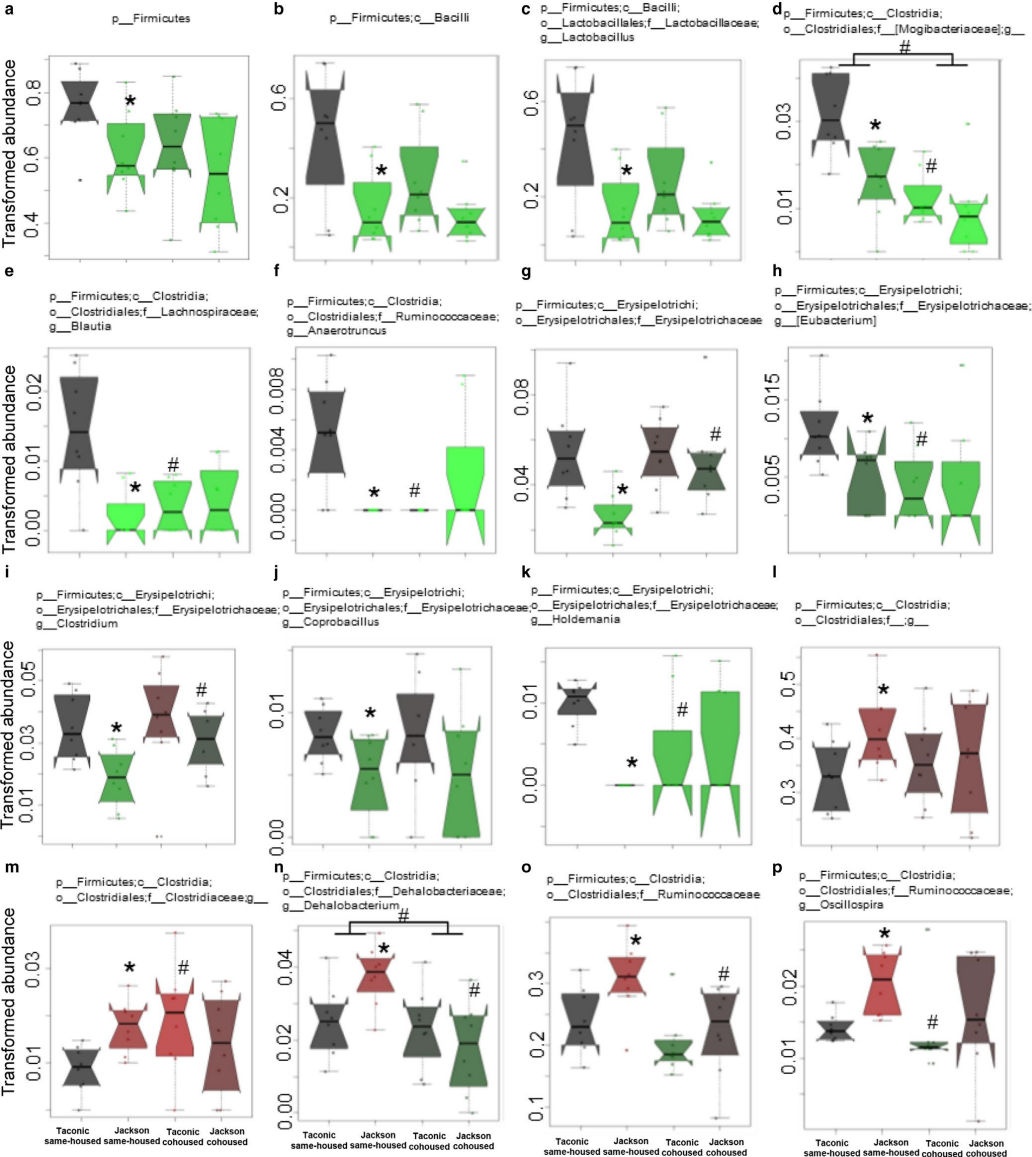
Effect of vendor on taxa within the Firmicutes phylum. In each case, the relative abundance data of bacteria in fecal DNA is shown for male Taconic and Jackson mice that were housed from weaning under same‐housed or cohoused conditions. The trapezoid boxes indicate the 25th and 75th percentile. The whiskers indicate the minimum and maximum values, and each dot denotes one mouse. The Tukey's notches on either side of the median line indicate within‐sample variance. Shown are the relative abundance of (a) total Firmicutes, (b) Bacilli, (c) *Lactobacillus*, (d) Mogibacteriaceae, (e) *Blautia,* (f) *Anaerotruncus*, (g) Erysipelotrichaceae, (h) *Eubacterium*, (i) *Clostridium*, (j) *Coprobacillus,* (k) *Holdemania,* (l) Clostridiales, (m) Clostridiaceae, (n) *Dehalobacterium,* (o) Ruminococcaceae, and (p) *Oscillospira.* *n* = 8 per group. *p < .05 and *q* < .25 versus Taconic mice; ^#^ *p* < .05 and *q* < .25 versus same‐housed mice. Statistical analysis was performed using MaAsLin (Morgan et al., 2012) on arcsine‐square root transformed relative abundance data

**Figure 7:**
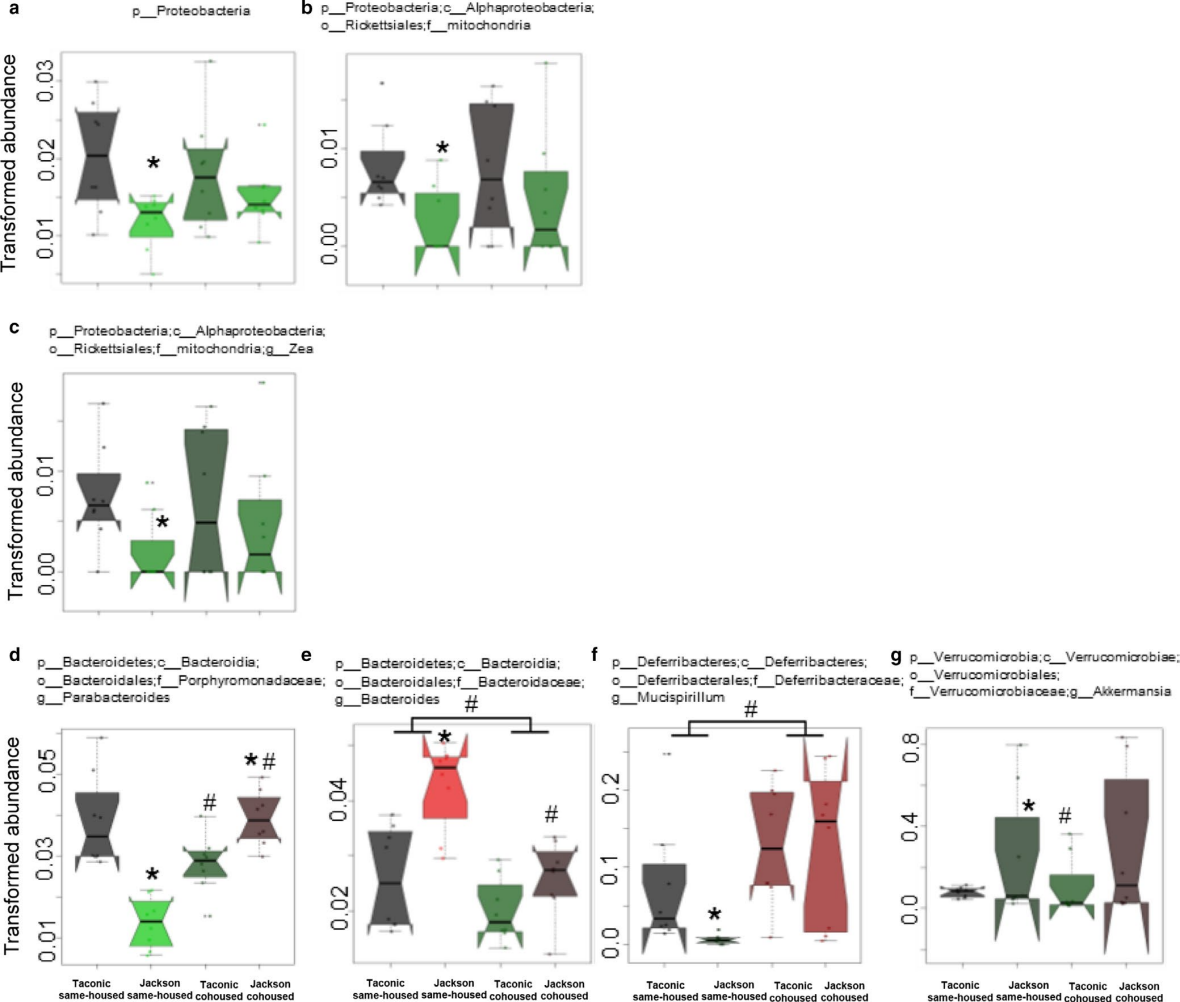
Effect of vendor on taxa within other phyla. In each case, the acrsine‐square root transformed relative abundances of bacteria in fecal DNA is shown for male Taconic and Jackson mice that were housed from weaning under same‐housed or cohoused conditions. Data are presented as described in Figure 5. Shown are: (a) total Proteobacteria, (b) Mitochondria, (c) *Zea*, (d) *Parabacteroides*, (e) *Bacteroides*, (f) *Mucispirillum*, and (g) *Akkermansia*. *n* = 8 per group. *p < .05 and *q* < .25 versus Taconic mice; ^#^ *p* < .05 and *q* < 0.25 versus same‐housed mice

To determine which bacterial taxa were different in Taconic and Jackson mice, we performed statistical analysis using MaAsLin (Morgan et al., 2012). The relative abundances of 23 taxa differed significantly in Taconic versus Jackson same‐housed mice (Figures 6 and 7). Bacteria within the Firmicutes phylum comprised the majority of these taxa (Figure 6). The relative abundances of total Firmicutes as well as the abundances of bacilli, *Lactobacillus*, Mogibacteriaceae, *Blautia, Anaerotruncus*, Erysipelotrichaceae, *Eubacterium, Clostridium*, *Coprobacillus*, *Holdemania* (Figure 6) were each significantly decreased in Jackson versus Taconic same‐housed mice and the abundances of Clostridiales, Clostridiaceae*, Dehalobacterium*, Ruminococcaceae, and *Oscillospira* (Figure 6) were significantly increased in Jackson versus Taconic same‐housed mice. Total bacteria within the Proteobacteria phylum, as well as the relative abundances of two taxa within the Proteobacteria phylum, mitochondria and *Zea,* were significantly decreased in Jackson versus Taconic same‐housed mice (Figure 7a–c). There were also some significant changes in certain taxa within other phyla. Within the Bacteroidetes phylum, the abundance of *Parabacteroides* was significantly decreased and the abundance of *Bacteroides* was significantly increased in Jackson versus Taconic same‐housed mice (Figure 7d and e). Within the Deferribacteres phylum, the abundance of *Mucispirillum* was significantly decreased (Figure 7f) and within the Verrucomicrobia phylum, the abundance of *Akkermansia* was significantly increased in Jackson versus Taconic same‐housed mice (Figure 7g), consistent with reports of others (Velazquez et al., 2019). Remarkably, the abundance of only 1 taxon, Parabacteroides, was significantly different in Taconic versus Jackson cohoused mice (Figure 7d). The data strongly support the existence of differences in the gut microbiomes of Jackson versus Taconic same‐housed mice and indicate that those differences were largely ablated when the mice were cohoused.

**Figure 8:**
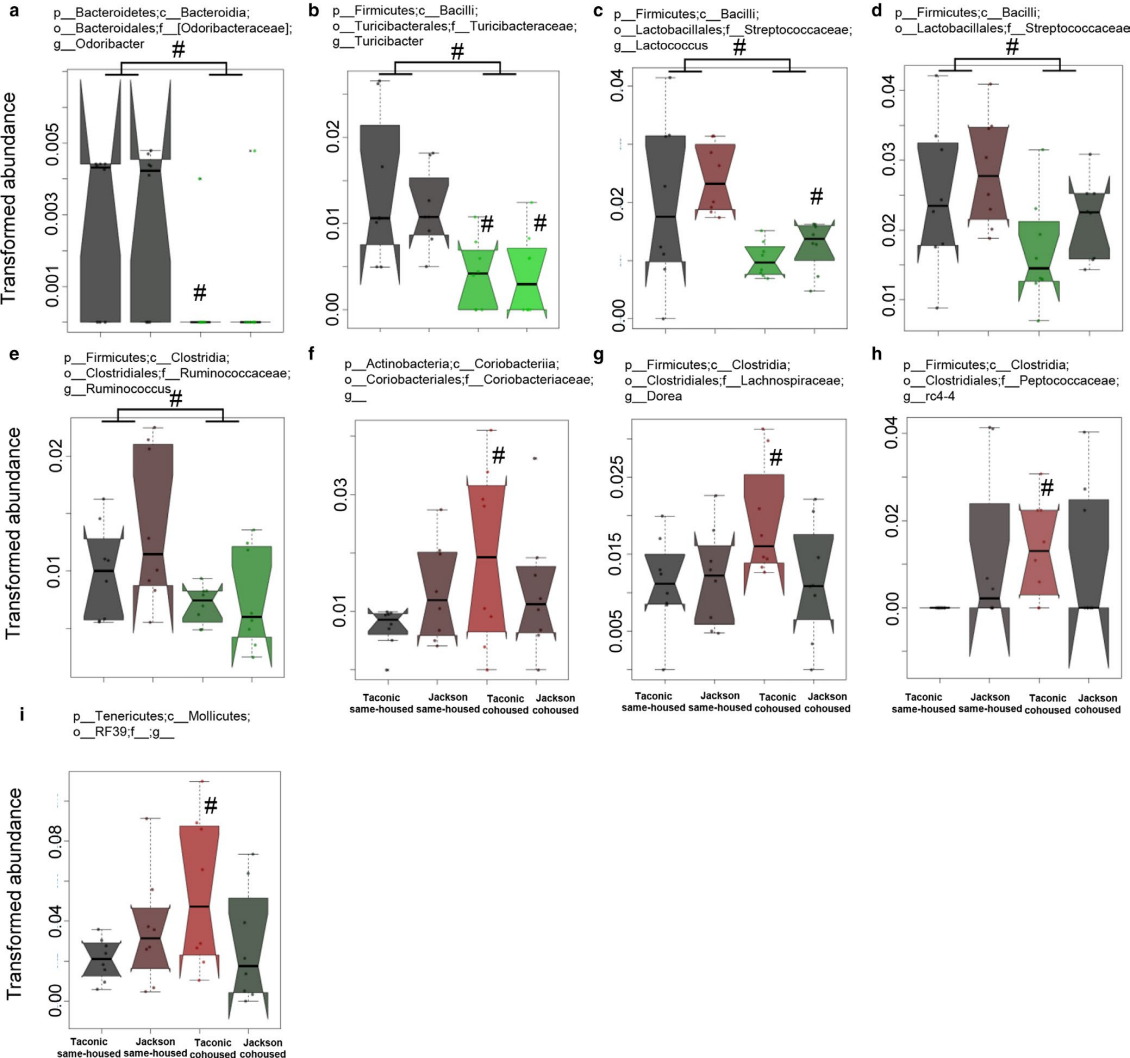
Additional taxa affected by cohousing. In each case, the arcsine‐square root transformed relative abundances of bacteria in fecal DNA is shown for male Taconic and Jackson mice that were housed from weaning under same‐housed or cohoused conditions. Data are presented as described in Figure 5. Shown are: (a) Coriobacteriaceae, (b) *Odoribacter*, (c) *Turicibacter*, (d) *Dorea*, (e) *rc4‐4*, (f) RF39, and (g) *Lactococcus*, and (h) Streptococcaceae. *n* = 8 per group. ^#^ *p* < .05 and *q* < .25 versus same‐housed mice

Differences in pulmonary responses to ozone were observed between same‐housed and cohoused mice rather than between Jackson and Taconic mice (Figures 2 and 3). Therefore, we sought to determine which bacterial taxa might associate with these cohousing‐related differences in the response to ozone. To do so, we used MaAsLin to compare same‐housed and cohoused mice. Compared to same‐housed mice*, Bacteroides* (Figure 7e), Mogibacteriaceae (Figure 6d), *Dehalobacterium *(Figure 6n), *Odoribacter *(Figure 8a), *Turicibacter *(Figure 8b), *Lactococcus *(Figure 8c), Streptococcaceae (Figure 8d), and *Ruminococcus *(Figure 8e) were significantly decreased and *Mucispirillum *(Figure 7f) were significantly increased in cohoused mice whether the mice were Taconic or Jackson. Other taxa were significantly different in same‐housed versus cohoused Jackson mice or in same‐housed versus Taconic mice, but not in both Taconic and Jackson mice (Figures 6, 7, 8).

**Figure 9:**
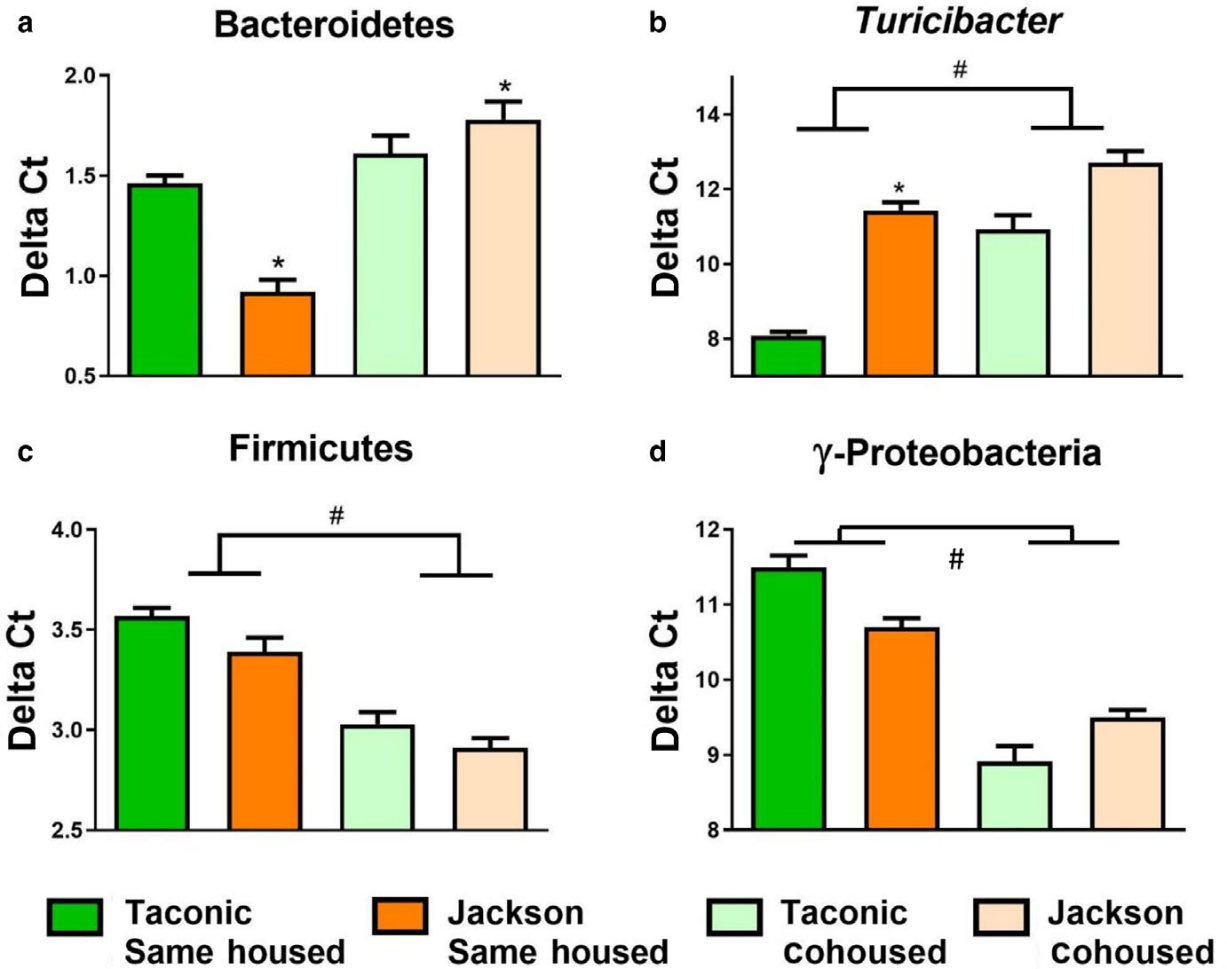
Effect of vendor and housing on bacterial taxa in fecal DNA from female mice. Shown are the abundances of (a) Firmicutes, (b) γ‐Proteobacteria, and (c) *Turicibacter* measured by qPCR of fecal DNA and normalized using pan‐bacterial primers. Fecal pellets were collected immediately before exposure to air or ozone. Data are mean ± *SE*. *n* = 9–22/group. * *p* < .05 versus Taconic same‐housed mice; ^#^ *p* < .05 for same‐housed versus cohoused mice

Whereas effects of cohousing on responses to ozone were observed in male mice, no such effects were observed in female mice (Figures 2 and 3). For that reason, we performed 16S rRNA sequencing only in the male mice. Nevertheless, we used qRT‐PCR to confirm that there were still differences in the gut microbiomes of the female Jackson versus Taconic same‐housed mice, and that effects of cohousing were also observed in the females. For example, qPCR assay indicated greater abundance (lower delta Ct) of Bacteroidetes and lower abundance (greater delta Ct) of *Turicibacter* in fecal DNA from Taconic than Jackson single‐housed female mice (Figure 9a and b). qPCR also indicated effects of cohousing on the abundance of *Turicibacter,* Firmicutes, and γ‐Proteobacteria (Figure 9b–d) in female mice.

## 4 DISCUSSION

The purpose of this study was to examine the effect of early life perturbations of the gut microbiome on responses to ozone later in life. To do so, we used mice from two vendors: The Jackson Laboratories and Taconic Farms. The gut microbiomes of these mice are known to differ (Ivanov et al., 2009; Velazquez et al., 2019). We housed weanling mice either with other same sex mice from the same vendor or with mice from the opposite vendor. Because mice are copraphagic, cohousing mice transfers fecal microbiota from one mouse to another mouse in the same cage. Indeed, our data indicate marked differences in the gut microbiomes of Jackson and Taconic same‐housed mice that were virtually abolished upon cohousing (Figures 5, 6, 7). The data validate the ability of our housing protocol to cause perturbations of the gut microbiome. Importantly, our results also indicate that these early life perturbations attenuated pulmonary responses to O_3_ in male mice. Cohoused male mice not only had a different microbiome than same‐housed mice, but also had reduced ozone‐induced AHR and fewer neutrophils. (Figures 2 and 3). Interestingly, this effect was not observed in female mice. The data indicate a role for early life microbial perturbations in pulmonary responses to a nonallergic asthma trigger and also indicate sex differences in this role.

We have previously reported a role for the gut microbiome in pulmonary responses to O_3_ in lean male mice (Cho et al., 2018). When male C57BL/6 mice from Taconic Farms were treated with a cocktail of antibiotics or with certain individual antibiotics, O_3_‐induced AHR and neutrophil recruitment were reduced. Rearing male mice under GF conditions had similar effects. We now report that perturbing the microbiome at an early age also reduces O_3_‐induced AHR and neutrophil recruitment (Figures 2 and 3). It is notable that these reduced responses to O_3_ did not require a reduction in microbial load, as occurs with antibiotics and GF conditions, only an alteration in the gut microbial community (Figures 6, 7, 8). It is also interesting to note that pulmonary responses to O_3_ were not different in Jackson and Taconic same‐housed mice (Figures 2 and 3), despite substantial differences in their gut microbial community structures (Figures 5, 6, 7) and differences in their genetics (Mekada et al., 2009). The absence of differences in responses to O_3_ in the C57BL/6 mice from Jackson Labs and Taconic Farms despite differences in their genetics indicates that although genetics do impact responses to O_3_ (Bauer & Kleeberger, 2010), the genetic differences extant in the C57BL/6 mice from these two vendors are not in regions that affect responses to O_3_. Similarly, the data suggest that only certain bacteria or the metabolic products of those bacteria contribute to pulmonary responses to O_3_. If so, alterations in those bacteria either by treatment with antibiotics, by rearing under GF conditions, or as a result of cohousing would all be expected to reduce responses to O_3_. In contrast, if those key bacteria were not among the bacteria that differed in same‐housed Jackson and Taconic mice, no differences in the response to O_3_ would be expected between these strains under same‐housed conditions, as observed. We do not know exactly which bacteria account for these effects, but among the bacterial taxa that were affected by cohousing, only *Odoribacter*, *Turicibacter*, *Lactococcus*, Streptococcaceae, *Ruminococcus*, Coriobacteriaceae, *Dorea*, *rc4‐4*, and RF39, were not also affected by vendor (Figure 8).

Whereas cohousing Jackson and Taconic mice from weaning had a significant effect on pulmonary responses to O_3_ in male mice, there was no effect in female mice (Figures 2 and 3), even though differences in the gut microbiomes of cohoused and same‐housed mice were observed in females (Figure 9) as well as in males (Figures 5, 6, 7, 8). There are sex differences in the gut microbiome (Org et al., 2016), and it may be that the bacteria whose alterations contribute to effects of cohousing on responses to O_3_ in male mice are already similarly altered by female sex. Consistent with this hypothesis, we and others have reported greater increases in airway responsiveness after O_3_ exposure in male than female mice (Birukova et al., 2019; Cho, Abu‐Ali, et al., 2019; Kasahara et al., 2019). Indeed, in both the Jackson and Taconic same‐housed mice in this study, O_3_‐induced AHR was greater in males than females (Figure 2). Moreover, we have reported that treating mice with a cocktail of antibiotics abolishes sex differences in the response to O_3_ (Cho, Abu‐Ali, et al., 2019), indicating a role for the microbiome in these sex differences.

There are several strengths to this study. First, we considered the possibility that some social stress might be induced in the cohoused mice by moving the pups into a cage with mice from another litter. We controlled for any possible effects of this early social stress on subsequent responses to O_3_ by also placing the same‐housed mice in cages with non‐sibling mice. Second, we initiated the cohousing protocol at weaning. At weaning, the gut microbiome of the pups is already undergoing substantial changes as a result of the change in diet from mother's milk to solid food (Hansen et al., 2012; Lee & Gemmell, 1972). Initiating the cohousing protocol at this time of flux in the microbial community structure may have permitted the establishment of the new microbiome in the cohoused mice (Figures 6, 7, 8). Finally, we studied both male and female mice, whereas studies of the effect of early life changes in the microbiome on allergic airway responses have evaluated only one sex (Trompette et al., 2014). As discussed above, our data emphasize the importance of considering both sexes in any study examining the role of the microbiome on host responses.

Our study also has some weaknesses. First, we examined the mice at early adulthood. We did so to permit sufficient time after initiation of the caging protocol to permit transfer of microbiota among cage mates and the establishment of a new microbiome. Our data (Figures 5, 6, 7, 8) indicate that this strategy was successful. However, we do not know whether the effect of cohousing would persist into later life or for how long. Second, we cannot rule out the possibility that changes in the lung or oral rather than the gut microbiome accounted for the observed effects of cohousing on pulmonary responses to O_3_. We examined the gut microbiome because our previously published data (Cho et al., 2018) indicate effects of the gut rather than the lung microbiome on responses to O_3_. In lean male mice, oral vancomycin reduces O_3_‐induced AHR (Cho et al., 2018) even though it affects the gut (Cho et al., 2018) but not the lung microbiome (Barfod et al., 2015).

Exactly how the microbiome alters pulmonary responses to O_3_ remains to be established. Current concepts suggest bacterial metabolites generated within the gut are able to cross the intestinal epithelium, enter the portal circulation, and are carried in the blood to distal organs like the lungs where they can impact function. For example, we have reported that gut bacterial fermentation of dietary fiber to short‐chain fatty acids (SCFAs) may be involved (Cho et al., 2018): antibiotic regimens that inhibit O_3_‐induced AHR also inhibit serum concentrations of SCFAs and exogenous administration of SCFAs augments responses to O_3_ in male mice. The observation that housing‐related changes in O_3_‐induced AHR and neutrophil recruitment were associated with similar changes in BAL concentrations of CCL2 (Figure 4a) suggests that microbial‐related effects on this chemokine may also be contributing. Interestingly, ligands that activate the SCFAs receptors, GPR41 and GPR43, inhibit CCL2 expression in epithelial cells (Kobayashi et al., 2017), suggesting that these events may be linked. A metabolic analysis also indicates differences in the impact of O_3_ on serum concentrations of bile acids, long chain fatty acids, and the polyamines spermine and spermidine in antibiotic‐ versus vehicle‐treated male mice (Cho, Osgood, Bell, Karoly, & Shore, 2019). Each of these biochemicals has the capacity to impact both airway responsiveness and neutrophil recruitment and could be contributing to the role of the microbiome in pulmonary responses.

## 5 CONCLUSION

Our data indicate that altering the gut microbiome early in life by cohousing Jackson and Taconic mice also altered subsequent pulmonary responses to O_3_ in male but not female mice. The data emphasize the importance of considering sex as a biological variable in studies of the effects of O_3_ on the lung and emphasize the need for strict attention to housing conditions and potentially vendor sourcing in mouse models of nonallergic asthma. More importantly, our data suggest that microbiome‐based therapeutics may ultimately prove useful in the treatment of nonatopic forms of asthma, but suggest that such therapies will need to be tailored differently for males and females.

## CONFLICT OF INTEREST

The authors have no conflicts of interest to disclose.
